# Anti-inflammatory action of geniposide promotes wound healing in diabetic rats

**DOI:** 10.1080/13880209.2022.2030760

**Published:** 2022-02-07

**Authors:** Xiao-yan Chen, Wen-wen Jiang, Yan-ling Liu, Zhao-xia Ma, Jian-qiang Dai

**Affiliations:** School of Medicine, Jiangxi University of Technology, Nanchang, China

**Keywords:** Diabetic skin lesion, interleukin-10, fasting plasma glucose, glycated haemoglobin

## Abstract

**Context:**

As a major active iridoid glycoside from *Gardenia jasminoides* J. Ellis (Rubiaceae), geniposide possesses various pharmacological activities, including anti-platelet aggregation and anti-inflammatory action.

**Objectives:**

This study explores the effect of geniposide in diabetic wound model by anti-inflammatory action.

**Materials and methods:**

Diabetic rodent model in Wistar rats was induced by streptozotocin combined with high-fat feed. The selected rats were divided into control group, the diabetic model group and geniposide subgroups (200, 400 and 500 mg/kg), and orally administrated once daily with saline or geniposide. Wound area and histochemical indicators were measured on day 7 after continuous administration, to assess lesion retraction, inflammatory cells and fibroblasts.

**Results:**

Geniposide notably enhanced lesion retraction by 1.06–1.84 times on day 7 after surgical onset in diabetic rats (*p* < 0.05). In the pathological experiment by HE staining, geniposide significantly reduced inflammatory cell infiltration and proliferation of fibroblasts in the central lesion regions. In diabetic rats treated with geniposide, the levels of pro-inflammatory factors (tumour necrosis factor-α (TNF-α), interleukin-1β (IL-1β)) and IL-6 were significantly reduced (*p* < 0.05), followed with the increment of IL-10 in a dose-dependent manner. The IC_50_ of geniposide on TNF-α, IL-1β and IL-6 could be calculated as 1.36, 1.02 and 1.23 g/kg, respectively. It assumed that geniposide-induced IL-10 expression contributed to inhibiting the expression of pro-inflammatory factors.

**Discussion and conclusions:**

Geniposide promoted diabetic wound healing by anti-inflammation and adjusting blood glucose. Further topical studies are required to evaluate effects on antibacterial activity and skin regeneration.

## Introduction

Diabetic skin ulcer (DSU) is a common complication of diabetes patients induced by high glucose (Capodanno and Angiolillo 2010). Most DSU patients will eventually progress to chronic and incurable ulcers. Different from ordinary skin lesion, nerve damage and the lesions of the surrounding blood vessels in diabetes patients make DSU difficult to heal, and complicated with repeated infections, all of which seriously affects quality of life (Lim et al. 2017). Due to many factors such as high costs, treatment tolerance and side effects, the treatment options of DSU on the market are still very limited.

*Gardenia jasminoides* J. Ellis (Rubiaceae) is a classic Chinese herb used for medicine and food; it is the main composition of many Chinese medicine prescriptions, such as Huanglian Jiedu decoction, Zhihong ointment and Zhi-zi-chi decoction. In Chinese medical theory, *Gardenia jasminoides* could reach meridians of heart, lung, and triple burner and suppress the evil fire, relieve internal heat, and cool blood in the body (Liu et al. 2013). Geniposide is a major iridoid glycoside in *Gardenia jasminoides* (Chen, Chen et al. 2020; Chen, Li et al. 2020). It has been reported in the literature that geniposide performed anti-inflammation and neuroprotective activities via modulating mitogen-activated protein kinase (MAPK)/nuclear factor kappa-B (NF-κB) and phosphatidylinositide 3-kinases-protein kinase B (PI3K-Akt) signalling pathways (Suzuki et al. 2001; Wu et al. 2019; Yuan et al. 2020; Zhou et al. 2020). In our earlier studies, the antithrombotic effect and anti-platelet aggregation of geniposide and genipin were also evaluated in animal models (Zhang et al. 2013; Liu, Chen, et al. 2021; Liu, Wang, et al. 2021). However, the potential effect of geniposide in wound healing of diabetic rats was not studied. This study evaluates the effect of geniposide in diabetic wound model by anti-inflammatory action.

## Materials and methods

### Animal and chemical agents

Healthy male Wistar rats 8 weeks old, body weight 250 ± 20 g were obtained from the Experimental Animal Center of Jiangxi University of Traditional Chinese Medicine; geniposide (HPLC > 95%, catalogue no. syz090911 from the Chengdu Dicotyledon Chinese Medicine Resources Co., Ltd., Chengdu, China); streptozotocin (catalogue no. S0130, Sigma-Aldrich, St. Louis, MO); and IL-6, interleukin-1β (IL-1β), tumour necrosis factor-α (TNF-α), IL-10 detection kit from R&D Systems (Minneapolis, MN) (IL-6: catalogue no. DY506; IL-1β: catalogue no. DY501; TNF-α: catalogue no. DY510; IL-10: catalogue no. SR1000). All animal experiment operations were approved and implemented by the Ethics Committee of Jiangxi University of Science and Technology (SCXK-2018-103).

### Preparation of diabetic wound model

All rats were continuously fed with high-fat diets (w/60% kcal from fat, 5.10 kcal/g, mainly containing 31.7% lard, 25.8% casein, 16.2% maltodextrin, 8.9% sucrose, 6.5% powdered cellulose, 3.2% soybean oil, 2.1% potassium citrate and others, catalogue no. 58Y1; TestDiet, St. Louis, MO) for 10 weeks, with free excess to food and water. Subsequently, rats were administrated intraperitoneally with a single injection of streptozotocin (55 mg/kg STZ in buffered-solution (pH 4.5) according to our experience). This protocol was modified according to former literature (Magalhães et al. 2019; Chen TJ et al. 2020). Two weeks after injection, the level of blood glucose was tested. Rats with fasting plasma glucose (FPG) more than 16.7 mmol/L were included as successful diabetic model (Beserra et al. 2019). The blood sugar levels of the rats were measured by using a OneTouch^®^ blood glucose meter (LifeScan, Wayne, PA). Glycated haemoglobin (HbA1c) was tested by kits from Chongqing Biospes Co., Ltd. (Chongqing, China).

For modelling of diabetic wound, diabetic rats were anaesthetized intraperitoneally with 1% sodium pentobarbital (40 mg/kg). After carefully cleaning the depilated area with 75% sterile medical alcohol, a hole puncher was used to create a 2 cm diameter wound reaching the lower fascia layer on the right side of back, and the sterile gauze was used to wrap the wound. The percentage of the wound area was calculated by the following formula: retraction (%)=(initial lesion area – area of lesion when measuring)/initial lesion area × 100 (Champeau et al. 2021).

### Experimental grouping and intervention

The selected rats were divided into control group (normal rats), the model group (diabetic rats treated with saline) and geniposide subgroups (diabetic rats treated with 200 mg/kg geniposide (Gen-L), 400 mg/kg geniposide (Gen-M) and 500 mg/kg geniposide (Gen-H)) for the study of skin lesion in diabetic rats. A total of 95 diabetic rats were used in these experiments. The dose range of geniposide was selected according to the maximum resolution of geniposide and our previous experience in animal studies. They were orally administrated with saline or geniposide solution (dissolved in saline) once daily for seven days after preparing the rat model of diabetic wound. The levels of FPG and HbA1c in each rat were measured on day 7 after continuous administration. The sampling collection was performed 1 h after last administration.

### HE staining

The method was performed according to the previous report with a few modification (Haghshenas et al. 2019). After intervention of geniposide for seven days, the marginal and intermediate tissues of skin lesion were taken, cleaned and fixed with 4% paraformaldehyde. After fixing in 4% paraformaldehyde at 4 °C for 24 h, the tissues were subjected to dehydration, embedding and sectioning with 5 μm thickness before HE staining. The indicators were consisted of the infiltration of inflammatory cell and the number of fibroblasts on the longitudinal section of the wound tissue under the microscope. Four fields of view at the central lesion regions were randomly selected for each sample to observe the changes under a 400-fold microscope. Since many neutrophils will move to the lesion region within first a few days, the number of inflammatory cells was identified by the nucleus stained in deep blue. The fibroblasts were recognized by cell morphology in dark purple.

### Detection of inflammatory factors in wound tissue

On day 7 after surgery, the wound tissue was extracted and homogenized on ice to prepare the homogenate, centrifuged at 12,000 rpm for 15 min and supernatant was collected for ELISA assay. ELISA kits (R&D Systems, Inc., Minneapolis, MN) were used to detect the levels of inflammatory factors such as IL-6, IL-10, IL-1β and TNF-α in the tissue homogenate.

### Statistical methods

The GraphPad Prism software 7.0 (La Jolla, CA) was used for statistical analysis, and the data were expressed in the form of mean ± standard error of the mean (SEM), and the statistical results *p* < 0.05 were considered as statistically significant.

## Results

### Effect of geniposide on blood glucose level in diabetic rats

**Figure 1.**
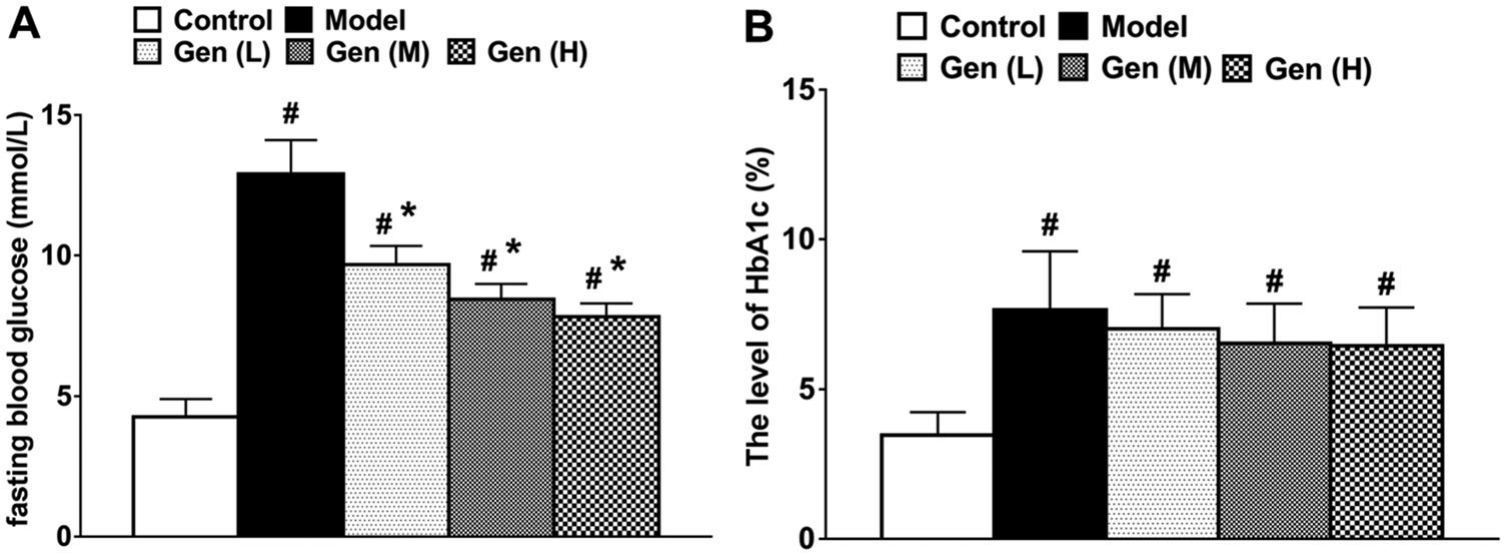
The levels of FBG (A) and HbA1c (B) in non-diabetic and diabetic rats were determined on day 7 after continuous administration. Data are shown as means ± SEM (*n* = 6 in each group with total of 30 rats). ^#^*p*< 0.05 and *p< 0.05 denote statistical significance compared with normal control group and the model group, respectively, analysed by one-way ANOVA followed by the *post hoc* Student–Newman–Keuls test.

In all diabetic rats, the blood glucose levels increased significantly. After oral administration of geniposide, the blood glucose level significantly decreased on day 7 after continuous administration compared to the model group (Figure 1, *p* < 0.05), whilst the level of HbA1c was not changed in treatment subgroups.

### Assessment of wound healing in diabetic rats

**Figure 2.**
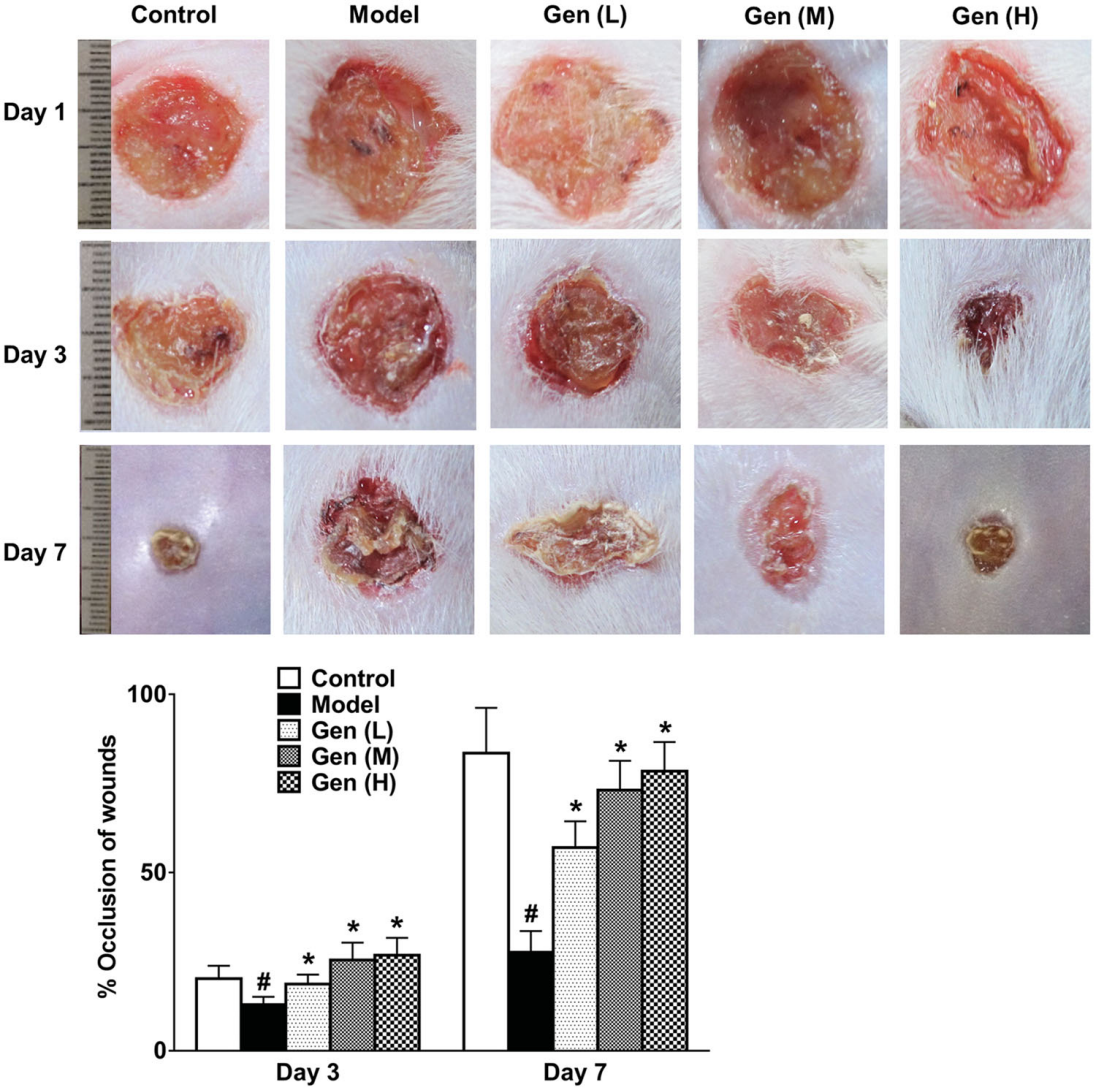
Geniposide promoted wound healing dose-dependently on day 1, 3 and 7 after continuous administration. Data are shown as means ± SEM (*n* = 4–5 in each group with total of 23 rats). ^#^*p*< 0.05 and *p< 0.05 denote statistical significance compared with normal control group and the model group, respectively, analysed by one-way ANOVA followed by the *post hoc* Student–Newman–Keuls test.

Results showed that diabetic rats received geniposide by gavage could significantly promote lesion retraction compared to the model group on day 3 and day 7 after surgery, respectively (Figure 2, *p* < 0.05). This improvement continued until the termination of treatment strategy.

### Assessment of skin lesion in diabetic rats by HE staining

**Figure 3.**
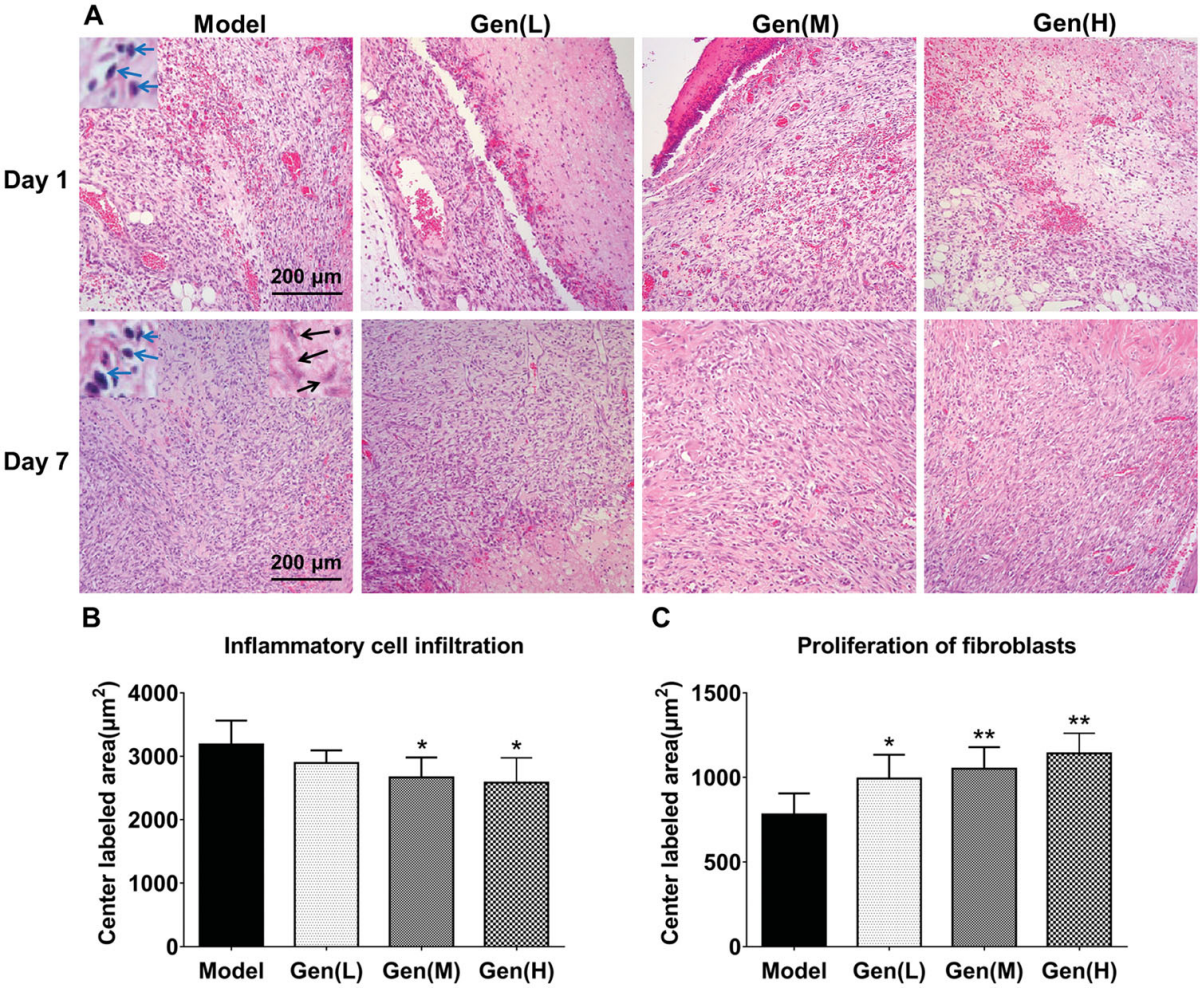
Geniposide promoted wound healing in HE staining via the regulation of inflammatory cell infiltration and proliferation of fibroblasts in the central lesion regions. (A) HE staining of skin wound in diabetic rats in each group. (B, C) The quantification of inflammatory cell infiltration and proliferation of fibroblasts was performed on day 7 after surgery and analysed by GraphPad Prism software (La Jolla, CA). The blue arrows indicated the infiltrated inflammatory cells, whilst the black arrows represented fibroblasts. Data are shown as means ± SEM (*n* = 4–5 in each group with total of 19 rats). *p< 0.05 and **p< 0.01 denote statistical significance compared with the model group, analysed by one-way ANOVA followed by the *post hoc* Student–Newman–Keuls test.

As shown in HE staining, the inflammatory cells and the proliferation of fibroblasts in the central lesion regions were quantified on day 7 in each sample (Figure 3(A–C)). The results showed that oral administration of geniposide (Gen-M and Gen-H) could significantly restrain the expression of inflammatory cells in the tissue by 16.4% and 18.9% (*p* < 0.05), and notably promote the proliferation of fibroblasts by 27.0%, 34.5% and 46.0% (*p* < 0.05).

### The changes of inflammatory factors in wound tissues

**Figure 4.**
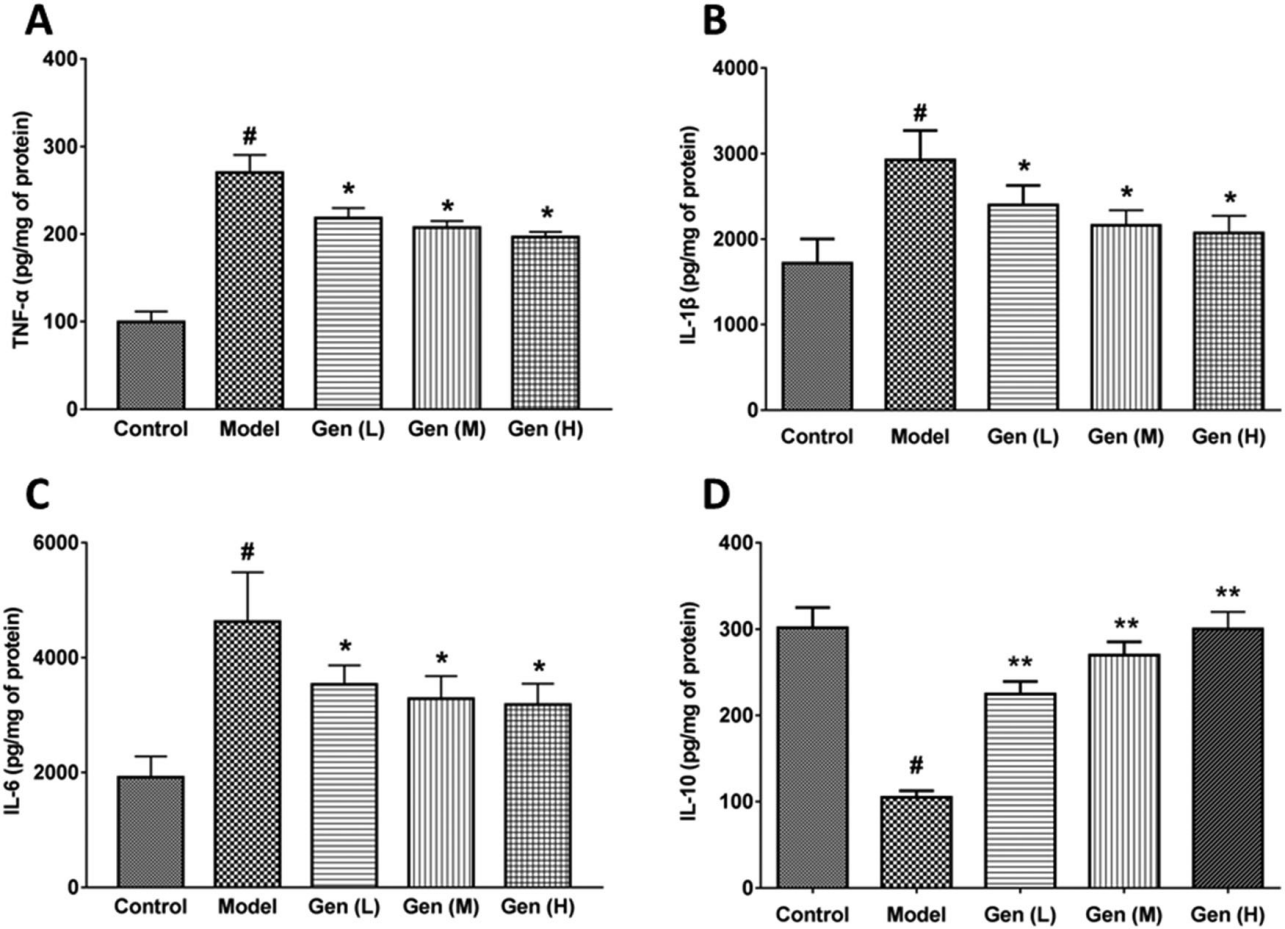
Geniposide inhibited the inflammatory factors in wound tissue. (A–D) The levels of pro-inflammatory factors (TNF-α, IL-1β), IL-6 and anti-inflammatory factor IL-10 were measured on day 7 in each group by ELISA kits. Data are shown as means ± SEM (*n* = 4–5 in each group with total of 23 rats). ^#^*p*< 0.05, *p< 0.05, and **p< 0.01 denote statistical significance compared with normal control group and the model group, respectively, analysed by one-way ANOVA followed by the *post hoc* Student–Newman–Keuls test.

The anti-inflammatory and pro-inflammatory factors in the wound tissue were detected by the ELISA method. We found that the skin lesion in diabetic rats was susceptible to infection due to the wound exposure, and the levels of TNF-α, IL-1β and IL-6 in wound tissues were significantly increased on day 7 after surgery. After oral administration of geniposide, the levels of pro-inflammatory factors (TNF-α, IL-1β) and IL-6 were significantly reduced (*p* < 0.05, Figure 4(A–C)), followed with the increment of anti-inflammatory factor IL-10 in a dose-dependent manner (Figure 4(D)). The IC_50_ values of geniposide on TNF-α, IL-1β and IL-6 were calculated as 1.36, 1.02 and 1.23 g/kg, respectively. The expression of IL-10 was enhanced by geniposide dose-dependently, with the increase of 1.14, 1.57 and 1.86 times (*p* < 0.01). We assumed that the anti-inflammatory action of geniposide mainly attributed to the increment of IL-10 level, which suppressed the expression of pro-inflammatory factors.

## Discussion

The large number of inflammatory cells produced in wound tissue of diabetic patients may strongly limit the process of wound closure. It has been reported in the literature that wounds under hyperglycaemic conditions could prohibit the expression of anti-inflammatory cytokines (such as IL-10) and intensify the expression of pro-inflammatory cytokines (such as TNF-α, IL-1β and IL-6) (Feng et al. 2016). In addition, pro-inflammatory cytokines not only participate in the inflammatory process, but also are considered as mediators of cell proliferation and differentiation. In activated stratum corneum cells, IL-6 stimulates the formation of granulation tissue, and promotes the occurrence of epithelial mechanisms and angiogenesis (Finnerty et al. 2006). TNF-α is shown to possess a dual role in reducing the formation of granulation tissue and the arrangement of collagen fibre (Breder et al. 2020). The anti-inflammatory factor IL-10 is a pleiotropic cytokine that exerts immunosuppressive or immunostimulatory effects in a variety of cell types. In addition, IL-10 can indirectly promote fibroblast proliferation and collagen deposition at the wound tissue (Wei et al. 2020).

Our results demonstrated that the inflammatory cells in the wound of diabetic rats were inhibited after oral administration of geniposide, especially in the central area of the lesion. Geniposide regulated these inflammatory mediators by reducing pro-inflammatory cytokines (such as IL-6) and up-regulating the level of anti-inflammatory cytokine IL-10. Inhibitor of nuclear factor kappa-B kinase (IKKs)/NF-κB pathway is the classical inflammatory pathway to produce TNF-α, IL-6 and IL-1β. Geniposide-induced IL-10 could also prohibit inflammatory factors by promoting the B-cell lymphoma factor 3 (Bcl-3) expression to inhibit the activity of IKKs (Wu et al. 2018). Besides, Li et al. reported that geniposide alleviated renal dysfunction in diabetic nephropathy mice through adenosine 5′-monophosphate-activated protein kinase (AMPK)/sirtuin1 (SIRT1)/NF-κB pathway, followed with the decreased levels of TNF-α, IL-6 and IL-1β (Li et al. 2020). In the disorders of atherosclerosis and osteoarthritis, geniposide was also proved to alleviate inflammation by regulating miR-101/mitogen activated protein kinase phosphatase-1 (MKP-1)/p38 pathway (Chen et al. 2018; Cheng et al. 2019). The expressions of IL-1, TNF-α and NO were also greatly suppressed by geniposide.

Poor wound healing is one of the common complications of diabetes in clinical practice. The skin lesion of diabetic patients is susceptible to inflammation due to high sugar levels and dysfunction of native immunity, all of which easily contribute to DSU. The delayed wound healing in DSU commonly attribute to the changes of endogenous factors, including collagen synthesis, fibroblast proliferation and immunological regulation, etc. (Monteiro-Soares et al. 2020). Our results showed that oral administration of geniposide could negatively regulate the blood glucose level of diabetic rats and enhance wound healing of DSU by suppressing inflammation and promoting fibroblast proliferation. In further study, the potential promoting effect of geniposide in wound healing should be evaluated in topical preparations.

## Conclusions

Geniposide promoted diabetic wound healing by anti-inflammation and adjusting blood glucose. Further topical studies are required to evaluate effects on antibacterial activity and skin regeneration.
